# Anti-Idiotype DNA Aptamer Affinity Purification–High-Temperature Reversed-Phase Liquid Chromatography: A Simple, Accurate, and Selective Bioanalysis of Bevacizumab

**DOI:** 10.3390/molecules24050857

**Published:** 2019-02-28

**Authors:** Tomohiro Yamada, Taro Saito, Yutaka Shimizu, Kaori Tsukakoshi, Hideki Hayashi, Hajime Mizuno, Daiki Tsuji, Keisuke Yamamoto, Kunihiko Itoh, Toshimasa Toyo’oka, Kazunori Ikebukuro, Kenichiro Todoroki

**Affiliations:** 1 Laboratory of Analytical and Bio-Analytical Chemistry, School of Pharmaceutical Sciences, University of Shizuoka, Shizuoka 4228526, Japan; t.yamada.bunseki3@gmail.com (T.Y.); hmizuno@u-shizuoka-ken.ac.jp (H.M.); toyooka@u-shizuoka-ken.ac.jp (T.T.); 2 Department of Biotechnology and Life Science, Graduate School of Engineering, Tokyo University of Agriculture and Technology, Tokyo 184-8588, Japan; hanataro191110@gmail.com (T.S.); s184955q@st.go.tuat.ac.jp (Y.S.); k-tsuka@cc.tuat.ac.jp (K.T.); ikebu@cc.tuat.ac.jp (K.I.); 3 Laboratory of Pharmacy Practice and Social Science, Gifu Pharmaceutical University, Gifu 501-1196, Japan; hayashih@gifu-pu.ac.jp; 4 Laboratory of Clinical Pharmacology and Genetics, School of Pharmaceutical Sciences, University of Shizuoka, Shizuoka 4228526, Japan; d-tsuji@u-shizuoka-ken.ac.jp (D.T.); itohk@u-shizuoka-ken.ac.jp (K.I.); 5 Department of Pharmacy, Seirei Hamamatsu General Hospital, Hamamatsu 4300906, Japan; k-yamamoto1@sis.seirei.or.jp

**Keywords:** aptamer affinity purification, high-temperature reversed-phase liquid chromatography, immunoaffinity purification, bevacizumab

## Abstract

This study presents a simple, accurate, and selective bioanalytical method of bevacizumab detection from plasma samples based on aptamer affinity purification–high-temperature reversed-phased liquid chromatography (HT-RPLC) with fluorescence detection. Bevacizumab in plasma samples was purified using magnetic beads immobilized with an anti-idiotype DNA aptamer for bevacizumab. The purified bevacizumab was separated with HT-RPLC and detected with its native fluorescence. Using aptamer affinity beads, bevacizumab was selectively purified and detected as a single peak in the chromatogram. HT-RPLC achieved good separation for bevacizumab with a sharp peak within 10 min. The calibration curves of the two monoclonal antibodies ranged from 1 to 50 μg/mL and showed good correlation coefficients (r^2^ > 0.999). The limit of detection (LOD) and lower limit of quantification (LLOQ) values for bevacizumab were 0.15 and 0.51 μg/mL, respectively. The proposed method was successfully applied to the bioanalysis of the plasma samples obtained from the patients with lung cancer and may be extended to plan optimal therapeutic programs and for the evaluation of biological equivalencies in the development of biosimilars.

## 1. Introduction

At present, more than 70 therapeutic monoclonal antibodies (mAbs) have been approved in the United States and Europe for therapeutic purposes in the field of oncology, inflammation, and infectious diseases [1,2,3]. There is a continuous increase in the demand for the bioanalysis of therapeutic mAbs to evaluate drug efficacy and establish optimal treatment plans. Furthermore, the competition for biosimilar development after the patent expiration of infliximab has become fierce; therefore, a quick, simple, and highly accurate bioanalysis method for bioequivalence evaluation with the original drug is desirable [4,5]. For the bioanalysis of therapeutic mAbs, tryptic digestion–liquid chromatography tandem mass spectrometry (LC-MS-MS) methods and ligand-binding assays (LBAs) are the two most commonly used strategies that offer complementary information [6,7]. However, the selective discrimination of target drugs or their tryptic digests among large amounts of immunoglobulin (IgG) proteins in the blood that only differ in the sequence of complementarity-determining regions (CDRs) is an inherent difficulty associated with the presently available bioanalytical methods. According to the report by Heidi et al., the total recovery rate of mAbs from the plasma samples by a tryptic digestion–LC/MS/MS method was only 14%, and a loss of 72% was attributed to the pretreatment process [8]. Therefore, the use of immunoaffinity purification may be an effective pretreatment approach even with a tryptic digestion–LC/MS/MS method. In this direction, various affinity ligand molecules such as anti-drug antibody [9], protein A [10], and protein G [11,12] have been reported. The recent improvement in the performance of high-resolution mass spectrometers has allowed quantification of therapeutic mAbs treated with affinity purification as intact or Fab-fragmented forms without tryptic digestion [13,14,15]. We have reported an LC-based intact bioanalysis method for bevacizumab and infliximab using immunoaffinity magnetic purification [16]. With these methods, the results may differ owing to the quality and affinity of the antibodies used [17,18], differences between lots, the preservation state, and other factors. Furthermore, immunoaffinity purification requires a large amount of anti-idiotype antibodies for immobilization on a purification support, resulting in high cost.

With each methodology, the choice of affinity molecule that extracts target components with accuracy and allows good recovery of the target from clinical samples during pretreatment is the key for the success of any convenient, robust, and accurate analytical method. To fit such preconditions, we contemplated the use of DNA aptamers as a new class of affinity molecules.

Aptamers are single-stranded DNA or RNA molecules that bind to a wide range of molecules with high specificity and affinity [19,20]. DNA is chemically stable and may be cost-effectively supplied by chemical synthesis. Many reports have been published using DNA aptamers as affinity ligands for protein purification from plasma samples [21,22].

We have recently developed an anti-idiotype aptamer toward therapeutic mAb, bevacizumab, that selectively recognizes the CDR of bevacizumab [23,24]. The dissociation constant of this aptamer to bevacizumab was very low (ca. 130 nM), and it showed no binding to endogenous IgGs in human plasma or to other therapeutic mAbs (infliximab, trastuzumab, cetuximab, and tocilizumab).

**Figure 1:**
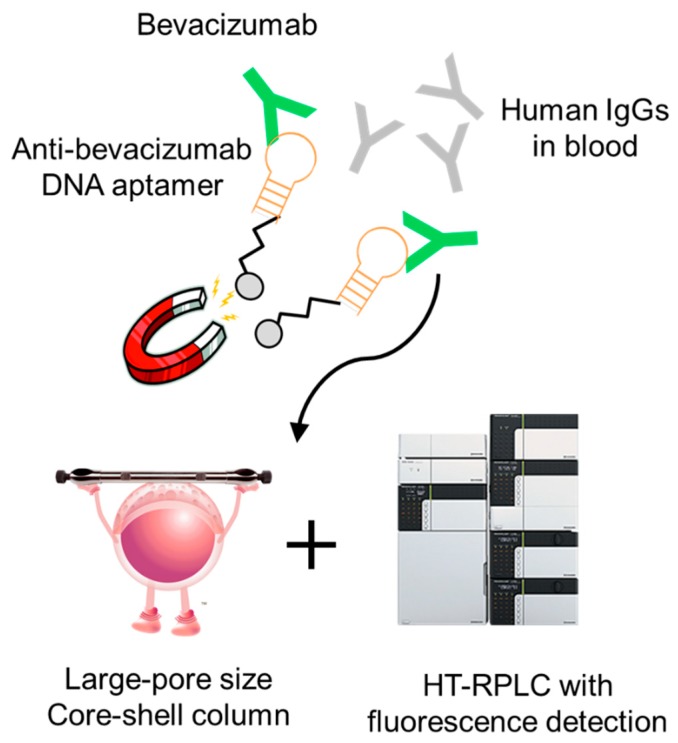
Schematic diagram of aptamer affinity purification–HT-RPLC for bevacizumab.

Here, we report a new affinity purification method for therapeutic mAbs using an anti-idiotype DNA aptamer, which could replace the conventional immunoaffinity methods. We further constructed a bioanalytical method for bevacizumab detection by combining this purification method and HT-RPLC. Figure 1 shows the schematic of our proposed method. Bevacizumab in blood samples was purified with magnetic beads immobilized with anti-idiotype DNA aptamer specific for bevacizumab. As a result, highly selective purification of bevacizumab is possible. The purified drug was separated by HT-RPLC using a core-shell column with a large pore size. The purified bevacizumab was separated by HT-RPLC and quantified with its native fluorescence. HT-RPLC, first reported by Dillon et al. [25], allows excellent separation of only IgGs from other remaining biological components as well as from other therapeutic mAbs [26]. This method does not require tryptic digestion or a highly expensive LC-MS/MS instrument.

The developed method was validated for sensitivity, linearity, accuracy, and precision. We successfully applied the method to the bioanalyses of plasma samples obtained from the patients with cancer that were administered the drug. Furthermore, we also compared the quantification results with those obtained with the previously described immunoaffinity purification method.

To the best of our knowledge, the present report is the first to describe an accurate and sensitive bioanalytical method for bevacizumab using aptamer affinity purification–HT-RPLC. Furthermore, the methodology using anti-idiotype DNA aptamer for the affinity purification of therapeutic mAbs is also reported for the first time.

## 2. Results and Discussion

### 2.1. HT-RPLC Separation of Bevacizumab

Bevacizumab purified by aptamer affinity magnetic beads was separated by HT-RPLC as previously optimized [16] and detected by its native fluorescence. As shown in the chromatograms described below, bevacizumab could be detected around 9 min as a sharp peak.

### 2.2. Selection of Affinity Support the Aptamer Affinity Magnetic Beads

We used Dynabeads as the aptamer affinity support based on the following three merits: uniform particle size and surface profile, good dispersibility, and selective collection with magnetism. The efficient binding of ligands prevented sample loss and led to good recovery and repeatability. Several types of Dynabeads with different functional groups for modification are available. Thus, we chose three types of beads (streptavidin, tosyl, and carboxylic acid) as affinity supports and evaluated the differences in their immobilization rate of DNA aptamer.

**Figure 2:**
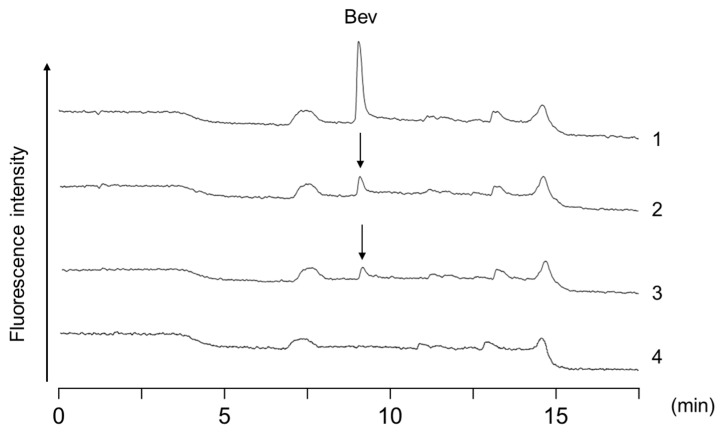
Chromatograms of residual solution when 100 μL of a 50 μg/mL bevacizumab standard solution was captured on the three types of aptamer affinity beads. Chromatograms: 1. bevacizumab standard solution; 2. tosylactivated beads; 3. carboxylic acid beads; 4. streptavidin beads. Peak: Bev: bevacizumab.

Figure 2 shows HT-RPLC chromatograms of a residual solution in response to the capture of 100 μL of a 50 μg/mL bevacizumab standard solution on three different aptamer affinity beads. At a high concentration of 50 μg/mL, bevacizumab was almost completely captured by streptavidin beads (>99.8%), whereas 75.8% and 85.5% of bevacizumab in the solutions were detected on the chromatograms without being captured on tosyl and carboxylic acid beads, respectively. Based on these results, we used Dynabeads streptavidin as the best affinity support in the following experiments.

### 2.3. Evaluation of Aptamer Affinity Purification

**Figure 3:**
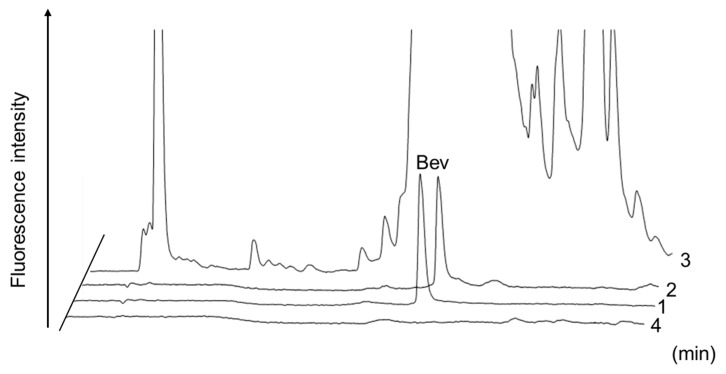
Chromatograms of the plasma samples spiked with 10 μg/mL of bevacizumab. 1. With aptamer affinity purification; 2. with aptamer affinity purification affinity beads by used three times; 3. without aptamer affinity purification; 4. with aptamer affinity purification of drug-free plasma. Peak: Bev: bevacizumab.

To confirm the specificity of the prepared aptamer affinity magnetic beads, plasma samples spiked with 10 μg/mL bevacizumab were purified with the beads, and their eluates were analyzed by HT-RPLC (Figure 3). With aptamer affinity purification, the peak for bevacizumab was not obscured by other components and was detected as a single and sharp peak (Chromatogram 1). Chromatogram 2 shows bevacizumab with aptamer affinity purification by affinity beads used three times. Recoveries at first and third uses of the beads were both high at 101.3% and 97.3%, respectively. This indicates the function of the aptamer was not lost by washing, and the affinity beads demonstrated high performance even in repeated use. For comparison, when analyzing the bevacizumab-spiked plasma sample without aptamer affinity purification, numerous peaks were detected in the chromatogram and obscured detection of bevacizumab (Chromatogram 3). Endogenous proteins and IgG molecules in human plasma could not be captured on the beads, so their corresponding peaks were not detected on the chromatograms (Chromatogram 4).

**Figure 4:**
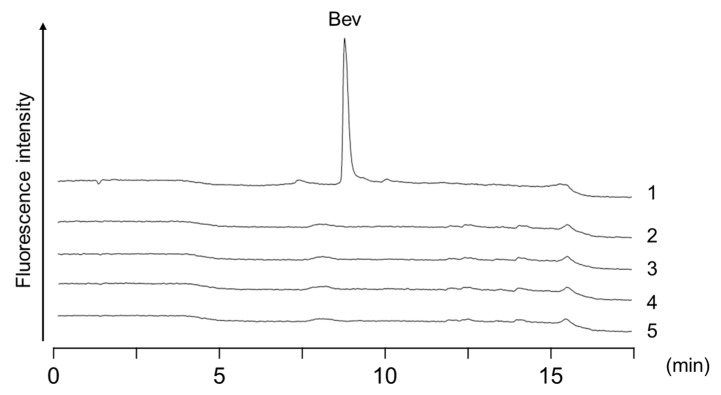
Chromatograms of 10 μg/mL of five therapeutic mAbs treated after aptamer affinity purification using anti-bevacizumab DNA aptamer. Chromatograms: 1. bevacizumab; 2. trastuzumab; 3. infliximab; 4. nivolumab; 5. tocilizumab.

We also evaluated the selectivity of these magnetic beads with other commercial mAb drugs (infliximab, trastuzumab, cetuximab, and tocilizumab) (Figure 4) and found that these aptamer affinity beads selectively captured bevacizumab from plasma samples as well as from therapeutic mAb preparations. The aptamer affinity beads could be reused at least five times after equilibration with a 100 mM sodium phosphate buffer (pH 7.4). In this treatment, a carry-over of target drugs was not observed in HT-RPLC analysis of blank plasma and drug-spiked (10 μg/mL) samples.

### 2.4. Method Validation

Different concentrations of bevacizumab ranging from 1 to 50 μg/mL with five replicates were chosen to draw calibration curves. The calibration equation, standard errors of the slope and the intercept were y = 49,859x + 21,801, 605.4, and 7097.5, respectively. The calibration curve of bevacizumab was observed between the peak area and drug concentration with good correlation coefficients (r^2^ > 0.999). This calibration curve range was wider than the range of 1–20 μg/mL in which we previously reported [16], owing to the high concentration of the affinity ligand immobilized on magnetic beads. According to the drug package inserts, the effective blood concentrations of bevacizumab ranged from 50 to 500 μg/mL [27]. The present method proved to cover this concentration range with simple sample dilution.

The limit of detection (LOD) and lower limit of quantification (LLOQ) values of bevacizumab were 0.15 and 0.51 μg/mL, respectively. The results of intra- and inter-day precisions, accuracy, and recovery of assays are listed in Table 1. The intra-day assay precisions of bevacizumab ranged from 2.3 to 5.5%, while the inter-assay precisions ranged from 3.0 to 6.2%; these values were within the acceptable limits. The bias was −4.9 to 14.2%. Recovery of bevacizumab ranged from 95.4% to 101.3%.

### 2.5. Determination of Bevacizumab in the Plasma Samples from Patients with Cancer

**Figure 5:**
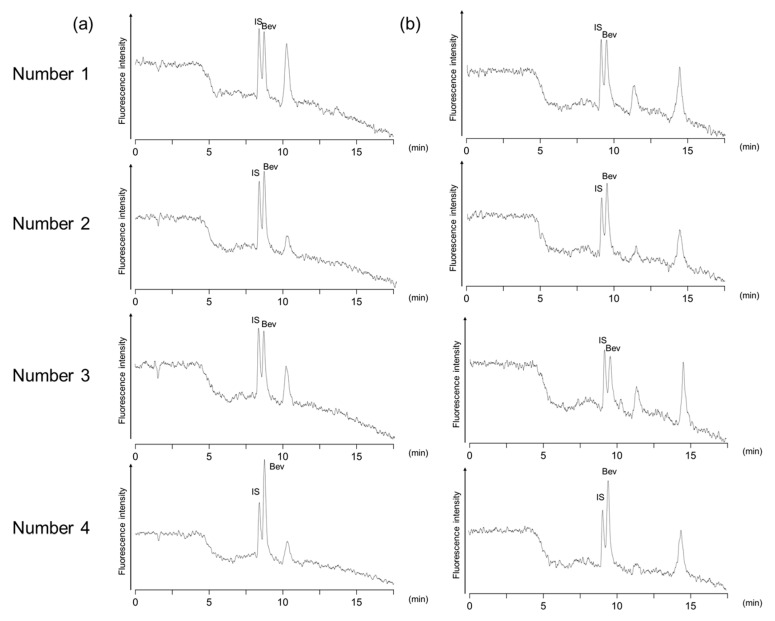
Chromatograms of plasma samples obtained from the patients with lung cancer that were administered bevacizumab with (**a**) aptamer affinity and (**b**) immune-affinity purification. Peaks: Bev: bevacizumab; IS: 20 μg/mL trastuzumab. Sample dilution ratios prior to affinity purification were 20-fold except for Patient 4 (25-fold) in (**b**).

We applied the proposed analytical method to the bioanalyses of plasma samples obtained from four male patients with lung cancer that were administered the drug. Furthermore, we also compared the quantification results with those obtained with the previously described immunoaffinity purification method. Clinical characteristics of the patients and plasma concentrations of bevacizumab analyzed by the present method and the immunoaffinity purification–HT-RPLC method is summarized in Table 2. Figure 5 shows the typical chromatograms of the plasma samples obtained from four male patients with lung cancer that were administered bevacizumab. Concentrations of bevacizumab in the samples were calculated from the ratio of the peak area using 20 μg/mL trastuzumab as internal standard (IS). If the analyzed concentration exceeded the calibration range, the plasma samples were appropriately diluted and re-analyzed. Clinical characteristics of patients and bevacizumab concentrations were analyzed by the present method and the immunoaffinity purification–HT-RPLC method [16]. Unlike the drug-spiked plasma sample, the patient samples showed several peaks in the chromatogram. However, the peak for bevacizumab was not obscured by other components, as evident from a single and sharp peak detected by both methods. Since a large amount of VEGF, which is a ligand of bevacizumab, presents in blood samples in cancer patients, autoantibodies against VEGF may also be produced. In the immunoaffinity purification that uses an anti-idiotype antibody, two peaks other than bevacizumab were detected, which is caused by recognition of the autoantibodies. On the other hand, since only one peak other than bevacizumab was detected in the aptamer affinity purification, it was considered that the difference between the aptamer and the anti-idiotype antibody in selectivity was reflected. Quantitative values measured by these two methods were in agreement and above the trough concentration (ca. 100 μg/mL) reported in a clinical analysis of bevacizumab at the same injected dose (every three weeks at 15 mg/kg) [27].

## 3. Materials and Methods

### 3.1. Reagents, Solutions, and Apparatus

Deionized and distilled water purified using the ELGA Purelab Flex system (ELGA, Marlow, UK) was used to prepare all aqueous solutions. LC-grade acetonitrile and isopropanol were purchased from Kanto Chemicals (Tokyo, Japan). Bevacizumab (Avastin^®^ 400 mg/16 mL for intravenous infusion), tocilizumab (ACTEMRA^®^ 80 mg for intravenous infusion), and trastuzumab (HERCEPTIN^®^ 150; 150 mg/7.2 mL for intravenous infusion) were produced by Chugai Pharmaceutical (Tokyo, Japan). Nivolumab (OPDIVO^®^ 100 mg for intravenous infusion) and infliximab (REMICADE^®^ for intravenous infusion 100) were procured from Ono Pharma (Osaka, Japan) and Mitsubishi Tanabe Pharma (Osaka, Japan), respectively. Dynabeads M-280 Streptavidin, Dynabeads M-280 tosylactivated, and Dynabeads M-270 carboxylic acid were obtained from Thermo Fisher Scientific (Waltham, MA, USA). Integrated DNA Technologies (Skokie, IL, USA) synthesized the 5′-biotinylated or aminohexyl anti-bevacizumab DNA aptamer (5′-GCGGTTGGTGGTAGTTACGTTCGC-3′). The anti-bevacizumab idiotype antibody was of enzyme-linked immunosorbent assay (ELISA) grade and obtained from Abnova Corporation (Taipei, Taiwan). Peptone from animal tissue was purchased from Sigma Aldrich (St. Louis, MO, USA), and bovine serum albumin (BSA), urea, sodium chloride, disodium hydrogen phosphate, and sodium dihydrogen phosphate were supplied by Wako Pure Chemical Industries (Osaka, Japan). Control human plasma was obtained from healthy volunteers at the University of Shizuoka (Shizuoka, Japan). All other chemicals were of the highest purity available and used as received.

### 3.2. Preparation of Aptamer Affinity Magnetic Beads Immobilized Anti-Bevacizumab DNA Aptamer

The 5′-biotinylated anti-bevacizumab DNA aptamer was coupled with the streptavidin-coated magnetic beads, while the 5′-aminohexyl anti-bevacizumab DNA aptamer was coupled with tosylactivated or carboxylic acid-coated magnetic beads.

The preparation of aptamer affinity beads using Dynabeads streptavidin was carried out as follows: Biotin-modified anti-bevacizumab DNA aptamer was diluted with a 100 mM Tris-EDTA (TE) buffer (pH 8.0) in advance at a final concentration of 100 μM (aptamer solution). A suspension of Dynabeads streptavidin (200 μL, 2 mg) was placed in a 1.5 mL polypropylene tube. After the removal of the supernatant, the remaining beads were washed with 300 μL of 100 mM phosphate-buffered saline (PBS), pH 7.4. After washing, 50 μL of an aptamer solution and 100 μL of 100 mM PBS (pH 7.4) were added to the beads. The mixture was vortexed at room temperature for 30 min with a microtube mixer (MT-360, TOMY SEIKO Corporation, Tokyo, Japan). After the removal of supernatant, the remaining beads were washed thrice with a washing buffer and treated with 100 μL of 100 mM PBS (pH 7.4) containing 0.1% BSA. The mixture was vortexed at room temperature for 3 h and, after the removal of supernatant, the remaining beads were washed thrice with a washing buffer. The beads were dispersed in 100 μL of a 100 mM sodium phosphate buffer (pH 7.4) and stored as a suspension at 4 °C.

Preparation of aptamer affinity beads using Dynabeads tosylactivated was performed as follows: A suspension of Dynabeads tosylactivated (66 μL, 2 mg) was placed in a 1.5 mL polypropylene tube and was washed thrice with 300 μL of 100 mM PBS. The beads were treated with 50 μL of a 100 μM 5′-aminohexyl anti-bevacizumab DNA aptamer solution in a 100 mM TE buffer (pH 8.0) at room temperature with vortexing. After addition of 50 µL of 3 M ammonium sulfate and 100 µL of PBS (pH 7.4), the mixture was vortexed at room temperature overnight.

Preparation of aptamer affinity beads using Dynabeads M-270 carboxylic acid was performed as follows: A suspension of M-270 carboxylic acid (66 μL, 2 mg) was placed in a 1.5 mL polypropylene tube and was washed thrice with 300 μL of a 25 mM 2-(*N*-morpholino)ethanesulfonic acid (MES) buffer (pH 4.8). The beads were treated with 50 μL of a 100 μM 5′-aminohexyl anti-bevacizumab DNA aptamer solution in a 25 mM MES buffer (pH 4.8), 10 µL of 1.25 M 1-ethyl-3-(3-dimethylaminopropyl)-carbodiimide (EDC) in a 100 mM MES buffer (pH 4.8) and 6 μL of a 25 mM MES buffer (pH 4.8). The solution was vortexed at room temperature overnight.

### 3.3. Evaluation of Aptamer Immobilization Amount on Three Different Magnetic Beads

We compared the amount of immobilized aptamers on three types of affinity beads prepared in Section 3.2. After the addition of 100 μL of 100 mM PBS (pH 7.4) containing 0.1% BSA to each magnetic bead type, the mixture was stirred at room temperature for 3 h and treated with 100 µL of a 50 μg/mL bevacizumab standard solution for 1 h with constant stirring. Unreacted bevacizumab was quantified by HT-RPLC from the ratio of the peak area of 20 μg/mL of the standard solution.

### 3.4. Isolation of Bevacizumab from Plasma Samples Using Aptamer Affinity Magnetic Beads

Affinity purification was executed using 2 mg of aptamer affinity magnetic beads per sample. After the removal of the solvent, the aptamer affinity beads were added to 100 μL of each plasma sample diluted by 10 times with a dilution buffer for mAbs (175 mM trehalose, 42 mM sodium dihydrogen phosphate, 8 mM disodium hydrogen phosphate, 0.4% Tween-20, pH 7.4) containing 0.1% peptone. The mixtures were vortexed and incubated at room temperature for 1 h. The beads were washed thrice with 100 μL of a washing buffer and the target mAbs were eluted once by incubation for 3 h with 100 μL of the mixture of 8 M urea and 3 M sodium chloride in a dilution buffer for mAbs (an elution buffer). After elution of the target mAb from aptamer affinity beads, the elute was collected and used as a sample for LC. For the analysis of patient samples, 50 μL of a 20 μg/mL trastuzumab (internal standard: IS) solution was added to this sample solution (50 μL). Aliquots of 2 μL were injected onto the LC-fluorescence system. After elution, the resulting beads were reused after washing thrice with 100 μL of 100 mM PBST (pH 7.4) and equilibration with 100 μL of 100 mM PBS (pH 7.4).

### 3.5. HT-RPLC System and Conditions

Most HT-RPLC conditions were repeated as previously described [16,26]. We used Prominence ultrahigh-performance liquid chromatograph system (Shimadzu, Kyoto, Japan) comprising a CBM-20A system controller, an SIL-20AC autosampler, two LC-20AD pumps, a DGU-20A online degasser, a CTO-20A column oven, an SPD-M20A photodiode array (PDA) detector, and an RF-20A fluorescence spectrometer equipped with a 12 μL flow cell. The fluorescence intensity was monitored at excitation and emission wavelengths of 278 and 343 nm, respectively. The collected data were analyzed using Lab Solutions LC (v. 1.21; Shimadzu).

An Aeris Widepore XB-C8 column, a core-shell-type analytical column, packed with 3.6 mm core-shell particles (150 × 2.1 mm I.D., Phenomenex, Torrance, CA, USA) was used. Mobile Phase A comprised water containing 0.1% trifluoroacetic acid (TFA), while Phase B was a mixture of 70% isopropanol, 20% acetonitrile, 9.9% water, and 0.1% TFA. Gradient profiling involved isocratic elution with A/B (90:10) for 1 min, linear gradient elution from A/B (90:10) to A/B (75:25) for 1 min, linear gradient elution from A/B (75:25) to A/B (50:50) for 13 min, isocratic elution with A/B (0:100) for 5 min, and isocratic elution with A/B (90:10) for 8 min. The flow rate of the mobile phase and the column temperature were set at 0.2 mL/min and 75 °C, respectively.

### 3.6. Preparation of Stock Solutions, Calibration Standards, and Quality Control Samples

Avastin injections were stored at approximately 4 °C and found to be stable for at least 6 months. These stock solutions were serially diluted with drug-free human plasma to obtain calibration standards at concentrations of 1, 5, 10, 20, 30, 40, and 50 μg/mL bevacizumab. For the preparation of quality control (QC) samples, a similar procedure was followed. Another stock solution was serially diluted in a different batch of drug-free human plasma to obtain quality control samples containing bevacizumab at concentrations of 1, 5, 10, 20, 30, 40, and 50 μg/mL. The validation sample at 100 μg/mL was prepared to assess the accuracy and precision after dilution in drug-free human plasma.

### 3.7. Method Validation

The proposed analytical method partially (intra- and inter-day precisions, accuracy, recovery) followed the FDA Bioanalytical method validation [28]. To obtain the validation parameters, peak areas were estimated by LabSolution, LC, and the baseline-to-baseline method was used for quantification.

#### 3.7.1. Precision

The precision of the assays was determined by the repeated measurement of six (bevacizumab; 1, 5, 10, 20, 30, 40, and 50 μg/mL) spiked QC samples. For intra-day precision, these levels were analyzed six times each day, whereas for inter-day precision, specimens of the spiked plasma samples at the same concentrations were analyzed three times per day for 3 days (*n* = 9), followed by the analysis of QC samples. The minimum acceptable precisions were <25% at 1 μg/mL and <20% at other concentrations.

#### 3.7.2. Accuracy

Accuracy was determined by the repeated measurement of three levels (1, 5, 10, 20, 30, 40, and 50 μg/mL; *n* = 3) of QC samples. The minimum acceptable bias was <25% at 1 μg/mL and <20% at other concentrations.

#### 3.7.3. Calibration Curve

For quantitative analysis, calibration standard solutions (*n* = 5) at concentrations ranging from 1 to 50 μg/mL (1, 5, 10, 20, 30, 40, and 50 μg/mL) were prepared by diluting the stock solutions. The equations of the calibration curves were determined using least-square linear prediction. Limit of detection (LOD) and lower limit of quantification (LLOQ) values were determined from signal-to-noise ratios of 3 and 10, respectively. For the analysis of patient samples, the calibration curve of bevacizumab was drawn from the peak area ratio between bevacizumab and IS [26].

#### 3.7.4. Recovery

The extraction recoveries for bevacizumab was evaluated by comparing the analytical results for spiked to non-spiked samples.

### 3.8. Plasma Sample Collection from Patients with Cancer Treated with Bevacizumab

Plasma samples of patients with lung cancer treated with bevacizumab (*n* = 4; age 55–69 years) were collected from Seirei Hamamatsu General Hospital (Hamamatsu, Japan). This study was approved by the Ethics Committees of Seirei Hamamatsu General Hospital (Approved No. 173) and the University of Shizuoka (Approved No. 26-49). Patients received periodic doses of Avastin as injections (every three weeks at 15 mg/kg). In this experiment, written informed consents were obtained from either the patients or their legal guardians after the purpose of this study was explained to them. Blood samples (5 mL) were collected from the treated subjects when they underwent biochemical examination of blood, and the times of drug administration were recorded.

### 3.9. Immunoaffinity Purification using RT-HPLC

Coupling of anti-bevacizumab idiotype antibodies to Dynabeads M-280 tosylactivated magnetic beads and isolation of bevacizumab from the plasma samples of patients were performed according to our previous method [16].

## 4. Conclusions

In this study, we developed a simple, accurate, and selective bioanalytical method for bevacizumab from plasma samples using anti-idiotype DNA aptamer affinity purification–HT-RPLC with fluorescence detection. Affinity purification with anti-bevacizumab aptamer allowed for the selective purification of bevacizumab from plasma samples with almost 100% recovery. The sensitivity, precision, and accuracy of the method were sufficient for the bioanalysis of the plasma samples from the patients with cancer. We successfully applied this method for the bioanalyses of plasma samples obtained from the patients with lung cancer. Unlike anti-idiotype antibodies, the chemically synthesized DNA aptamers are readily available as low batch-to-batch variation products, and various homogeneous bioanalyses using these aptamers can be performed. In addition, our aptamer affinity purification method may be used as a pretreatment process with the tryptic digestion–LC-MS/MS method. The method described herein is applicable to various fields such as planning optimal therapeutic programs and for the evaluation of biological equivalencies in the development of biosimilar.

## Figures and Tables

**Table 1:** Intra- and inter-day precision, accuracy, and recovery.

Spiked Concentration (μg/mL)	Precision (%)		Accuracy (Bias, %)	Recovery (%)
	Intra-Day	Inter-Day		
1	5.5	6.2	14.2	99.9
5	3.4	3.2	4.0	100.3
10	2.3	3.0	−2.9	101.4
20	3.2	3.1	−3.6	100.3
30	2.4	3.5	−0.9	99.3
40	2.5	3.4	−2.2	96.7
50	2.5	3.9	−4.9	95.4

**Table 2:** Clinical characteristics of four male patients with lung cancer and plasma concentrations of bevacizumab analyzed by the present method and the immunoaffinity purification–HT-RPLC method.

Number	Age	Height (cm)	Weight (kg)	Disease Status	Date of Administration and Blood Collection	Bevacizumab Concentration (μg/mL)	
						The Present Method	Immunoaffinity Purification–HT-RPLC [16]
1	58	166.0	70.1	IV	17/05/09 17/05/27	174.4	188.4
2	69	162.9	74.5	IV	17/03/14 17/04/04	205.1	202.2
3	65	174.6	66.5	IIB	17/05/09 17/06/01	172.9	171.1
4	55	173.0	71.2	IV	17/05/30 17/06/20	312.7	324.5
